# In vivo cloning of PCR product via site-specific recombination in *Escherichia coli*

**DOI:** 10.1007/s00253-024-13239-7

**Published:** 2024-06-29

**Authors:** Moein Aliakbari, Ali Asghar Karkhane

**Affiliations:** Department of Industrial and Environmental Biotechnology, National Institute of Genetic Engineering and Biotechnology (NIGEB), Tehran, Iran

**Keywords:** In vivo cloning, Site-specific recombination, CRISPR/Cas9, Direct transformation, Plasmid curing

## Abstract

**Abstract:**

Over the past years, several methods have been developed for gene cloning. Choosing a cloning strategy depends on various factors, among which simplicity and affordability have always been considered. The aim of this study, on the one hand, is to simplify gene cloning by skipping in vitro assembly reactions and, on the other hand, to reduce costs by eliminating relatively expensive materials. We investigated a cloning system using *Escherichia coli* harboring two plasmids, pLP-AmpR and pScissors-CmR. The pLP-AmpR contains a landing pad (LP) consisting of two genes (*λ int* and *λ gam*) that allow the replacement of the transformed linear DNA using site-specific recombination. After the replacement process, the inducible expressing *SpCas9* and specific sgRNA from the pScissors-CmR (CRISPR/Cas9) vector leads to the removal of non-recombinant pLP-AmpR plasmids. The function of LP was explored by directly transforming PCR products. The pScissors-CmR plasmid was evaluated for curing three vectors, including the origins of pBR322, p15A, and pSC101. Replacing LP with a PCR product and fast-eradicating pSC101 origin-containing vectors was successful. Recombinant colonies were confirmed following gene replacement and plasmid curing processes. The results made us optimistic that this strategy may potentially be a simple and inexpensive cloning method.

**Key points:**

*•The in vivo cloning was performed by replacing the target gene with the landing pad*.

*•Fast eradication of non-recombinant plasmids was possible by adapting key vectors*.

*•This strategy is not dependent on in vitro assembly reactions and expensive materials*.

**Supplementary Information:**

The online version contains supplementary material available at 10.1007/s00253-024-13239-7.

## Introduction

The insertion of heterologous DNA sequences into plasmids, particularly in studies that analyze the function of many genes, requires simple and inexpensive approaches. Traditional restriction-based methods due to partial digestion/ligation of DNA fragments, the limitations associated with internal cut sites, attachment of unwanted amino acids to the target protein, and several DNA purification steps are problematic for large-scale cloning projects (Ellis et al. 2011; Stevenson et al. 2013). Some of these problems have been solved by modified restriction-based methods, including Flexi® Cloning System (Blommel et al. 2006) and Golden Gate Assembly (Engler and Marillonnet 2014), which rely on rare-cutting and type IIS restriction enzymes, respectively.

Ligation-independent methods, such as LIC (Aslanidis and De Jong 1990), SLIC (Li and Elledge 2007), in-fusion seamless cloning (Zhu et al. 2007), and Gibson Assembly (Gibson et al. 2009), are based on creating 5′ or 3′ overhangs in DNA fragments with overlapping ends by the exonuclease activity of T4 DNA polymerase or T5 exonuclease. The circular plasmid can be obtained by complementary single-stranded annealing of DNA fragments and DNA polymerase’s gap-filling activity before the transformation. These methods enable the cloning of inserts without conventional time-consuming digestion/ligation reactions, but they define another type of costly materials and extra steps.

PCR-based cloning methods, including Restriction Free (Chen et al. 2000; Van Den Ent and Löwe 2006), Exponential Megapriming PCR (Ulrich et al. 2012), Recombination-Assisted Megaprimer (Mathieu et al. 2014), QuickStep (Jajesniak and Wong 2015), and Circular Polymerase Extension Cloning (Quan and Tian 2011), have been developed without restriction enzymes and ligases but often are performed with more than two primers. These approaches require complete-sequence amplification of the plasmid and insert. Accordingly, in addition to the potential complexity of the PCR strategy, the mutation probability will also increase. Unwanted mutations in the plasmid backbone can affect expression levels even though the insert sequence is correct (Ortega et al. 2019). In all mentioned protocols, template plasmids are eliminated by the *Dpn*I-treatment step to reduce colony background.

In vivo cloning methods allow the DNA fragments to be assembled inside the cell through recombination mechanisms. Despite simplicity and cost-effectiveness, the in vivo approaches have not been widely used for cloning applications. This may be due to their low efficiency since, occasionally, high efficiency is more important than affordability. However, the simple concept of in vivo cloning resulted in further research to improve its efficiency (Li et al. 2011; Jacobus and Gross 2015; Kostylev et al. 2015). The IVA (in vivo assembly) method has provided the possibility of all cloning procedures (insertions, deletions, mutagenesis, and sub-cloning) using a single universal protocol consisting exclusively of a single-tube PCR (García-Nafría et al. 2016). In addition, the in vivo cloning process has been investigated for accurately assembling up to three DNA fragments into plasmids up to 16 kb (Huang et al. 2017).

In vitro recombinational cloning methods, such as Gateway cloning technology, are suitable for high-throughput (HTP) cloning. The Gateway system (Walhout et al. 2000) is inspired by the site-specific recombination mechanism of the bacteriophage lambda. In this system, PCR fragments flanked by *attB* sites recombine with the donor vector’s *attP* sites by integration host factor (IHF) and λ integrase (BP reaction). The BP reaction forms the entry vectors containing fragments flanked by *attL* sites. In the next step, the entry vector’s *attL* sites recombine with the destination vector’s *attR* sites by λ integrase, λ excisionase, and IHF (LR reaction). The LR reaction forms the expression vectors containing fragments flanked by *attB* sites. Gateway vectors have the insertion site containing the lethal (*ccdB*) gene flanked by *att* sites and must be propagated separately in the CcdB-resistant strains. Therefore, after plasmid transformation in a CcdB-sensitive strain, vectors in which the lethal gene is not replaced with the target gene will lead to the host’s death (Hartley et al. 2000). Gateway cloning technology (Walhout et al. 2000) is a restriction-ligation-free (RLF) method that provides highly efficient cloning without the time-consuming screening for recombinant colonies. However, this system has two-step cloning and is expensive compared to many strategies due to its reliance on vectors, proteins (λ Int, λ Xis, and IHF), and CcdB-resistant strains (Ferigolo et al. 2022).

The CRISPR/Cas9 molecular scissors, known in many bacteria as an immune system against foreign plasmid or phage DNA (Doudna and Charpentier 2014), is a powerful tool for genome editing in eukaryotes and bacteria (Choi and Lee 2016; Li et al. 2020). This technology, in many cases, allows the fast and efficient curing of plasmids from their host. In the plasmid curing, *Cas9* nuclease by the specific gRNA, which forms a ribonucleoprotein (RNP), binds to a plasmid’s target sequence and creates a DNA double-strand break (DSB) which leads to its removal from the cell (Citorik et al. 2014; Lauritsen et al. 2017).

**Fig. 1:**
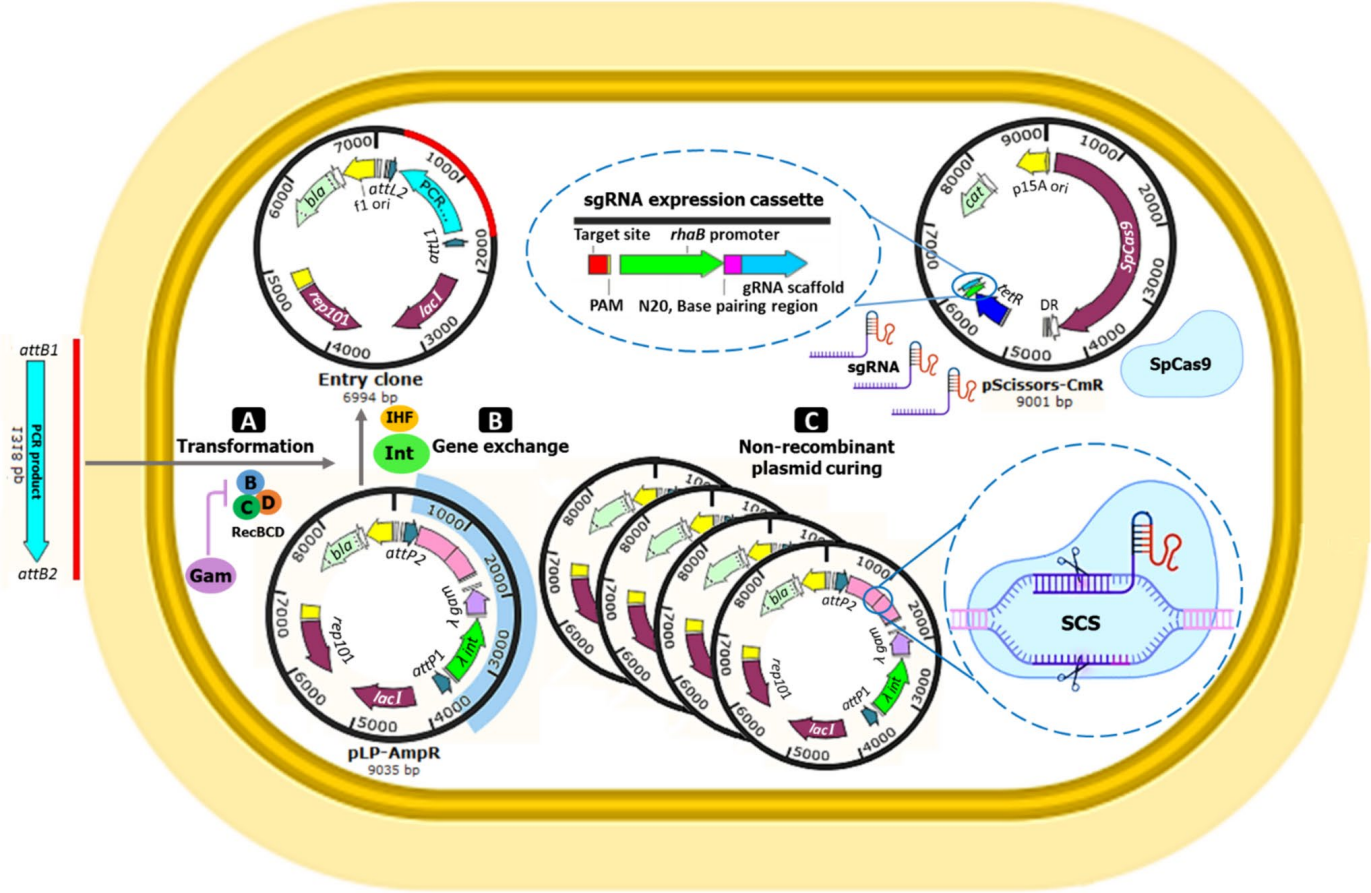
ICPS method. **A** The direct transformation of PCR product flanked by *attB* sites into the *E. coli* competent cells. Lambda Gam protein expressed by the landing pad of pLP-AmpR (light blue region) protects the linear DNA from degradation by V (RecBCD) and I (SbcB) host exonucleases. **B** *Int* and IHF proteins replace the PCR product with the LP to form a recombinant (entry) plasmid lacking the SCS sequence and resistant to double-strand break (DSB) caused by SpCas9. **C** Inducible expressing *SpCas9* and specific sgRNA from the pScissors-CmR leads to removing all vectors except the recombinant plasmids. Screening with CRISPR-Cas9 and ampicillin results in the death of plasmid-free cells; therefore, only ampicillin-resistant cells carrying the recombinant plasmid can grow

We have designed a molecular method called in vivo cloning of the PCR product by site-specific recombination (ICPS), aimed at simplifying gene cloning and reducing its costs. This plan neither requires expensive materials nor the relatively complex PCR strategy for amplifying DNA fragments with overlapping ends. Following the direct transformation of the PCR-amplified target gene into the host, all the requirements for obtaining the recombinant colony are provided in the cell. This *in vivo* cloning system consists of gene exchange and negative screening processes performed with the function of two plasmids in *Escherichia coli* BL21(DE3). A landing pad (LP) flanked by *att*P sites, including *two* genes (*λ int and λ gam*) and a SpCas9-cleavage sequence (SCS), has been inserted into the first plasmid (pLP-AmpR)*.* The Gam protein prevents linear DNA degradation by V (RecBCD) and I (SbcB) host exonucleases (Datsenko and Wanner 2000; Mosberg et al. 2010). After transforming the PCR product flanked by *attB* sites, the Int and IHF proteins replace this linear fragment with the LP. The non-recombinant plasmids are removed by the inducible coexpression of *Cas9* and specific gRNA in the second plasmid (pScissors-CmR). Using the ICPS, unlike time-consuming screening methods, only colonies with the recombinant plasmid survive after an efficient pre-selection by CRISPR-Cas9 and ampicillin screening (Fig. 1).

## Materials and methods

### Reagents, bacterial strains, and plasmids

Ampicillin (Amp, 100 μg/ml), chloramphenicol (Cm, 33 μg/ml), kanamycin (Kan, 30 μg/ml), and tetracycline (Tet, 10 μg/ml) were utilized separately or in combination for screening. Isopropyl-ß-D-thiogalactopyranoside (IPTG, 0.5 mM) was used to induce the expression of the *tetA* gene. Anhydrotetracycline (aTc, 200 ng/ml) and L-rhamnose (10 mM) were used to induce the *SpCas9* gene and gRNA, respectively. Strains and plasmids are listed in Table 1. *E. coli* DH5α was used for routine cloning processes, and *E. coli* BL21(DE3) was employed as an in vivo cloning system for gene exchange and plasmid curing experiments. Genomic DNA of *E*. *coli* XL1-Blue was used to PCR-amplify the tet repressor (*tetR*) gene (Table 1).

**Table 1:** Bacterial strains and plasmids used in this study

**Bacterial strain**	**Genotype**	**Reference**
*E. coli* BL21(DE3)	*fhuA2* [*lon*] *ompT gal* (λ DE3) [*dcm*] Δ*hsdS* λ DE3 *= λ sBam*HI*o* Δ*Eco*RI-B *int::*(*lacI::*PlacUV5*::T7 gene1*) i21 Δ*nin5*	(Studier and Moffatt 1986)
*E. coli* DH5α	F^-^, ϕ80d, *lacZ* ΔM15, *endA1*, *recA1*, *hsdR17* (rK-mK-), *supE44*, *thi1*, *gyrA96, relA1,* Δ*(lacZYA-argF)* U169	(Woodcock et al. 1989)
*E*. *coli XL1*-Blue	*recA1*, *endA1*, *gyrA96*, *thi1*, *hsdR17*, *supE44*, *relA1*, *lac* [F′ *proAB*, *lac*I ^*q*^, *lacZ* ΔM15, *Tn10* (*Tet* ^*R*^ )]	(Stratagene, La Jolla, CA, USA)
**Plasmid**	**Properties/description**	**Reference**
pUC19 pET-21b(+)	pMB1 origin, *ampR*, cloning vector T7 promoter, pBR322 origin, *ampR*, expression vector	(Yanisch-Perron et al. 1985) (Novagen, Madison, WI, USA)
pBR322	pMB1 origin, *ampR*, *tetR*, cloning vector	(Bolivar et al. 1977)
pGP1-2	*T7 RNA polymerase* gene under λ pL promoter, p15A origin, *kanR*	(Tabor and Richardson 1985)
pKD46	P_araB_ γ β exo (red recombinase), pSC101 origin, *ampR*	(Datsenko and Wanner 2000)
pCas9 pCDFDuet-SSEC pET-21b(+)-int-gam pET-21b(+)-SCS	*SpCas9* gene under native promoter, p15A origin, *cat* CloDF13 origin, *aadA*, synthetic sgRNA expression cassette (SSEC) pET-21b(+) containing the *attp1*, *λ int* and *λ gam* pET-21b(+) containing the *attP2* and *HSV-tk* with SCS	(Jiang et al. 2013) This study This study This study
pET-21b(+)-LP	pET-21b(+) containing the landing pad (*attP1*, *λ int*, *λ gam*, SpCas9-cleavage site (SCS), *attP2*)	This study
pLP-AmpR	Constructed by replacing the origin of pET-21b(+)-LP with pSC101	This study
pCas9-SSEC	pCas9 containing the SSEC	This study
pScissors-CmR	Constructed by insertion of a *tetR* gene in pCas9-SSEC and replacing SpCas9 native promoter with a Tet-inducible promoter	This study
pScissors-CmR-SCS	Constructed by insertion of the SCS in pScissors-CmR	This study
pGP1-2-SCS	Constructed by insertion of the SCS in pGP1-2	This study
pKD46-SCS	Constructed by insertion of the SCS in pKD46	This study

### Design of sgRNA expression cassette

The sgRNA expression cassette consisting of an L-rhamnose-inducible promoter (P_*rhaB*_), target-binding sequence (spacer), guide RNA scaffold, and termination signal was designed to direct the Cas9 nuclease to a specific location in target plasmids. Several highly efficient gRNA from the *HSV-tk* gene, which is not present in the host chromosome, by the web-based ChopChop tool (https://chopchop.cbu.uib.no/) was identified. After NCBI-BLAST, the final gRNA with minimum off-target binding to the *E. coli* BL21(DE3) chromosome was selected.

### Plasmid construction

Plasmids and designed primers are given in Tables 1 and 2, respectively. To gene exchange process, the genes of *λ int* and *λ gam* under the *J23119* promoters, *attP* sites, and a SpCas9-cleavage sequence (SCS) were synthesized in two separate plasmids of pET-21b(+)-int-gam and pET-21b(+)-SCS (Table 1). The SCS fragment digested by *Sal*I and *Eco*RI was subcloned into pET-21b(+)-int-gam to form a landing pad (LP) flanked by *attP* sites in the pET-21b(+) plasmid. The low copy number origin of pSC101 from pKD46 was PCR-amplified by F2172 and R2172 primers (Table 2). The pSC101 origin fragment was digested by *Bgl*I and used to replace the pET-21b(+)-LP’s origin to form pLP-AmpR, a very low copy number plasmid.

**Table 2:** Designed primers for this study

Name	Sequence (5′→3′)	Site	Amplified fragment
F2172	GCAGGACCACTTCTGCGCTC	*Bgl*I	pSC101 ori- Rep101
R2172	CAGTAGGCCGGCATGGCGAATCCATGGGTATGGACAG	*Bgl*I	
F702	ACTGATGTCGACTTGACAGCTAGCTCAGTCCTAGGTATAATTGTCAACAAAAATTAGGAATTAATG	*Sal*I	Tet repressor (*tetR*) gene
R702	GTTATGGTCGACTTAAGACCCACTTTCACATTT	*Sal*I	
F2676	TTTCGTCTAGATTGACATCCCTATCAGTGATAGAGATACTCATTATGGATTTAATTTAAACTT	*Xba*I	Part of the *SpCas9* gene
R2676	CCACGATTTTTATCAGAACG	*Mlu*I	
F1318	GGGGACAAGTTTGTACAAAAAAGCAGGCTGAAGGAGATAGAACCATGAAATCTAACAATGCGCT	*att*B1	*tetA* gene
R1318	GGGGACCACTTTGTACAAGAAAGCTGGGTTGGCTCCAATTCTTGGAGTG	*att*B2	
T7 promoter	TAATACGACTCACTATAGGG	*-*	Insert flanked by *attL* sites
T7 terminator	GCTAGTTATTGCTCAGCGG	*-*	

For plasmid curing experiments, the SCS sequence was subcloned into the pGP1-2 and pKD46 (Gene Bridges, Heidelberg, Germany) plasmids. Additionally, the vector pCas9 (42876; Addgene, Cambridge, MA, USA) was subjected to four modifications. (1) Insertion of the synthetic sgRNA expression cassette (SSEC): the SSEC sequence was subcloned into the plasmid pCas9 by using the *Sal*I and *Bgl*I restriction sites. (2) Replacement of the *SpCas9* promoter: the initial part of the *SpCas9* gene was amplified from the plasmid pCas9 by the primers of F2676, containing the combinatorial Tet-inducible promoter sequence (Cox III et al. 2007), and R2676 (Table 2). Then, this PCR product was replaced with the native *SpCas9* promoter of plasmid pCas9-SSEC by using the *Xba*I and *Mlu*I restriction sites. (3) Insertion of the *tetR* gene: the *tetR* gene was amplified from the *E. coli* XL1-Blue genome by the primers of F702, containing a constitutive *J23119* promoter sequence, and R702 (Table 2). Then, this PCR product was inserted into the plasmid pCas9-SSEC to generate the vector pScissors-CmR. (4) Subcloning of the SCS sequence: the SCS sequence was inserted into the vector pScissors-CmR for the self-plasmid curing experiments.

### Plasmid-curing experiments

The plasmid pCas9-SSEC was transformed into each of the BL21(DE3) hosts carrying either pET-21b(+)-LP, pET-21b(+), pGP1-2-SCS, pGP1-2, pKD46-SCS, or pKD46. Approximately 3 × 10^9^ colony-forming units per ml (CFU/ml) from an LB medium overnight culture (containing Amp+Cm or Kan+Cm) of these strains (except carrying pKD46-SCS) were inoculated in 10 ml LB broth supplemented with L-rhamnose and chloramphenicol at 32 °C under shaking of 180 rpm. Samples were taken after 8, 12, 16, and 24 h and diluted to a density of approximately 600 CFU/ml. Then, 100 μl of diluted samples was plated onto LB agar containing Amp or Kan. The plasmid pScissors-CmR was transformed into each of the BL21(DE3) hosts harboring either pLP-AmpR or pKD46. Additionally, to evaluate the self-curing of pScissors-CmR-SCS and the removal of pLP-AmpR simultaneously, the plasmid pScissors-CmR-SCS was transformed into BL21(DE3) carrying pLP-AmpR. Overnight cultures (containing Amp+Cm) of these strains were inoculated in 10 ml LB broth containing anhydrotetracycline and L-rhamnose with/without chloramphenicol at 32 °C under shaking of 180 rpm. Samples were taken after 8, 12, 16, and 24 h and diluted to a 600 CFU/ml density. Then, 100 μl of diluted samples was plated on LB agar containing Amp and Cm (each separately or in combination).

### In vivo gene exchange process

The F1318 primer (including the Shine-Delgarno element between the *attB1* site and the target gene sequence) and R1318 primer (containing *attB2* site at 5′ end) were designed for PCR amplification of the tetracycline-resistance gene (*tetA*) from the pBR322 plasmid (Table 2). A single colony of the BL21(DE3) strain harboring the pLP-AmpR plasmid was overnight cultured in 10 ml LB broth containing Amp at 30 °C under shaking at 180 rpm. The culture was inoculated (1:100 dilution) into 50 ml LB broth containing Amp. After growing to OD600 = 0.5, the electrocompetent cell was prepared by three steps of washing in a chilled 10% glycerol / dH_2_O solution. The mixture of cell suspension (100 μL with a density of 3 × 10^9^ CFU/ml) and agarose gel-purified PCR product digested by *Dpn*I (1 μg) was electroporated (25 μF, 200 ohms, 1.8 kV) and recovered in 900 μL of the super optimal broth with catabolite repression (SOC) medium at 32 °C under shaking of 220 rpm for 60 min. Finally, 3 × 10^9^ CFU/ml of cells were plated on LB agar supplemented with tetracycline and IPTG and were incubated at 30 °C for 24 h. The transformation efficiency of the cells was evaluated using 150 ng of pUC19.

### ICPS method

To achieve an optimized protocol, different parameters such as cell harvest time, cell density, and PCR product concentration were investigated. A single colony of the BL21(DE3) strain harboring pLP-AmpR and pScissors-CmR plasmids was overnight cultured in 10 ml LB broth containing Amp and Cm at 30 °C under shaking of 180 rpm. The culture was inoculated (1:100 dilution) into 500 ml LB broth containing Cm and incubated at 30 °C under shaking of 180 rpm. After growing to OD600s of 0.15, 0.25, and 0.5, cells were harvested by centrifugation at 4000 g for 10 min and prepared by three steps of washing in a chilled 10% glycerol/dH_2_O solution. The washed cells were diluted to different densities of 1.5, 2, 2.5, 3, and 3.5 (× 10^9^) CFU/ml. The mixture of cell suspension (100 μL) and agarose gel-purified PCR product digested by *Dpn*I (in different amounts of 0.5, 1, 1.5, and 2 μg) was electroporated (25 μF, 200 ohms, 1.8 kV) and recovered in 900 μL of the SOC medium at 32 °C under shaking of 220 rpm for 60 min. The elimination of non-recombinant plasmids was started by the transfer of recovered cells to 10 ml LB broth supplemented with anhydrotetracycline, L-rhamnose, and Cm for 12 h. Finally, the cells (3 × 10^9^ CFU/ml) were plated on LB agar containing Amp (200 μg/ml) and were incubated at 30 °C for 24 h.

## Results

**Fig. 2:**
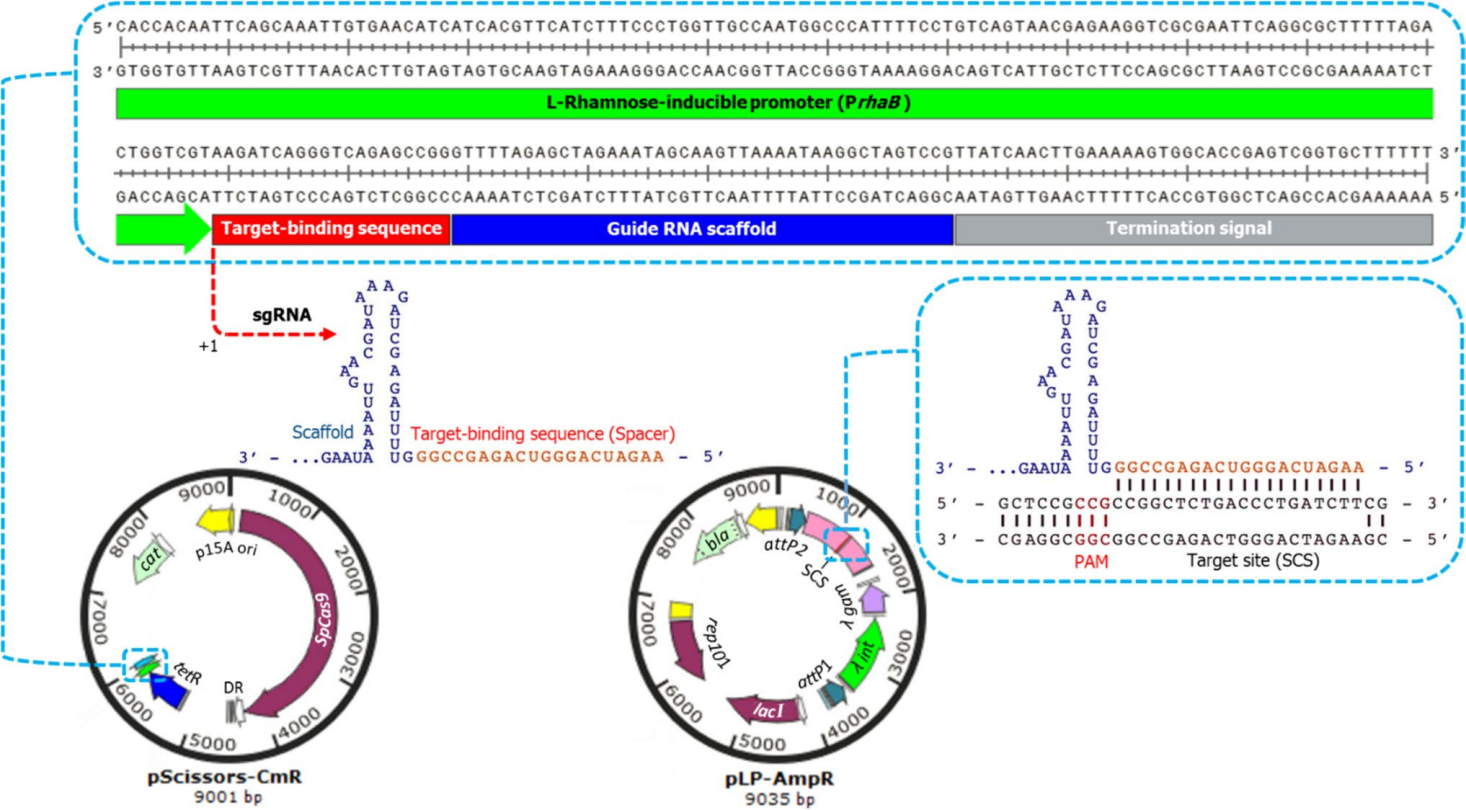
Sequence and function of the synthetic sgRNA expression cassette (SSEC). The SSEC consists of an L-rhamnose-inducible promoter (P_*rhaB*_), target-binding sequence, guide RNA scaffold, and termination signal. A sgRNA that expresses from SSEC of pScissors-CmR can bind to the target site (SCS) on pLP-AmpR. The designed gRNA could not lead to a DNA double-strand break (DSB) in the bacterial chromosome and cell death

**Fig. 3:**
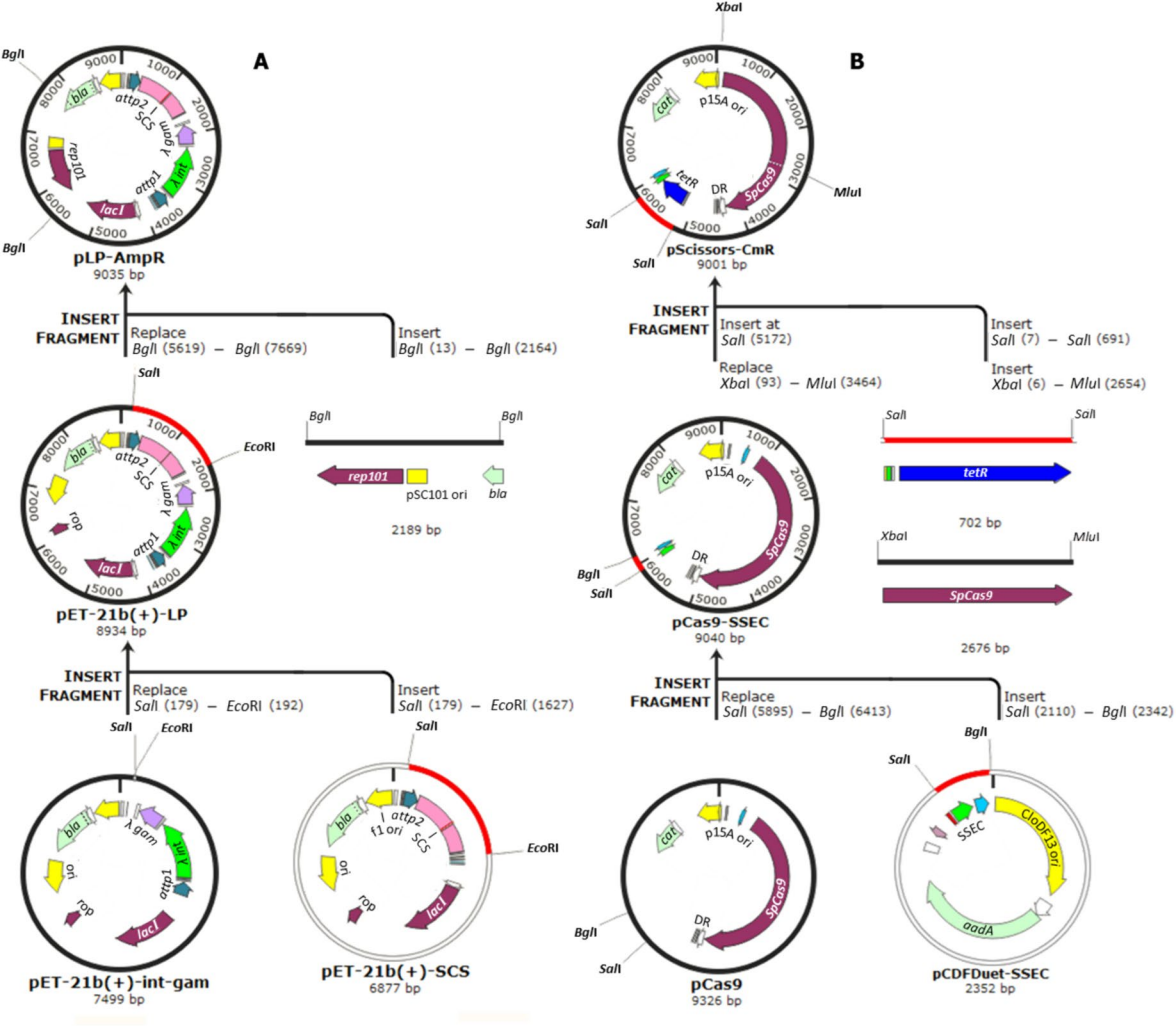
Construction and adaptation of the key vectors for gene exchange and plasmid curing experiments. A The genes of *λ int* and *λ gam* under the *J23119* promoter, *attP* sites, and a SpCas9-cleavage sequence (SCS), which were synthesized in two separate plasmids, were assembled as a landing pad (LP) flanked by *attP* sites in the pET-21b(+) plasmid. The pLP-AmpR, a very low copy number plasmid, was constructed by replacing the origin of pET-21b(+)-LP with a PCR-amplified pSC101 origin from pKD46. B The pScissors-CmR was created by applying three modifications to pCas9 (1) subcloning of a synthetic sgRNA expression cassette (SSEC) consisting of L-rhamnose-inducible promoter (P_*rhaB*_). (2) PCR amplification of the 2676 bp from the *SpCas9* gene containing a modified Tet-inducible promoter and replacement of this PCR product with the native *SpCas9* promoter. (3) Cloning of the *tetR* gene containing the constitutive *J23119* promoter

Inducing the SSEC by L-rhamnose in BL21(DE3) containing pCas9-SSEC showed that the designed gRNA could not lead to a DNA double-strand break (DSB) in the bacterial chromosome and cell death (Fig. 2). We constructed and adapted key vectors for gene exchange and plasmid curing experiments (Fig. 3). Plasmids were confirmed by enzymatic digestion analysis and sequencing.

**Fig. 4:**
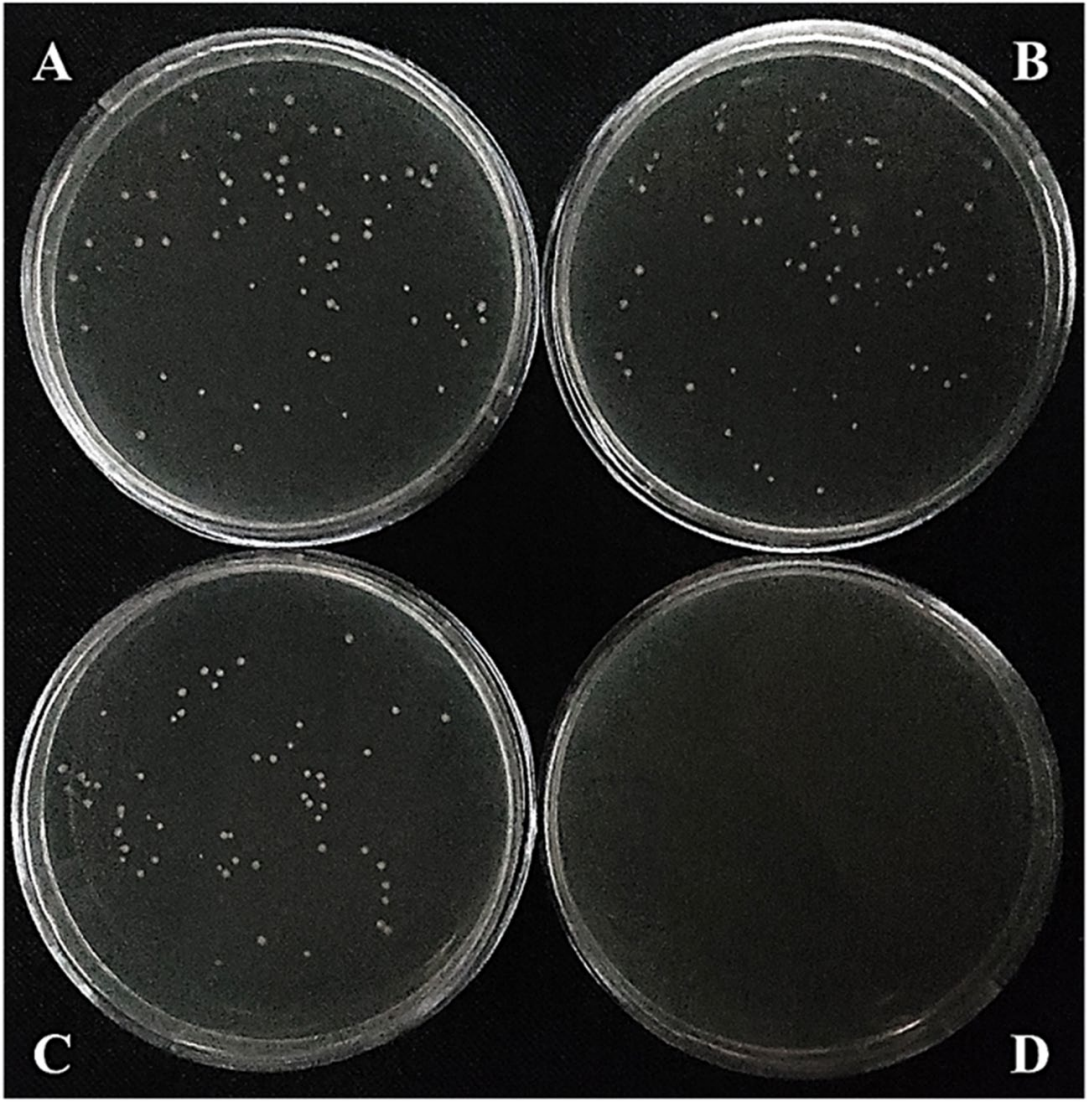
Plasmid curing by pScissors-CmR. A total of 100 μL of samples with a density of approximately 600 CFU/ml was plated on LB agar containing Amp after induction by anhydrotetracycline and L-rhamnose for 12 h. In each repetition of the protocol (a total of eight repetitions), the number of colonies obtained from the control samples (ranging from 51 to 78 colonies) was not significantly different. **A** BL21(DE3) harboring pKD46 (negative control) can be survivors without SpCas9 cleavage sequence (SCS) in the absence of the inducers and **B** in the presence of it, **C** the growth of BL21(DE3) harboring pLP-AmpR in the absence of the inducers, and **D** the death of it in the presence of the inducers

Plasmid curing by pCas9-SSEC in each of the BL21(DE3) hosts carrying either pET-21b(+)-LP, pGP1-2-SCS, or pKD46-SCS was studied in parallel with control strains containing either pET-21b(+), pGP1-2, or pKD46. The elimination process of pET-21b(+)-LP and pGP1-2-SCS was inefficient and only resulted in cell growth delay compared to control cultures for a few hours. In contrast, no growth of the strain harboring pKD46-SCS after subculturing compared to the control indicated that this plasmid was removed from the cells even without L-rhamnose induction. In addition, plasmid curing with the pScissors-CmR in BL21(DE3) harboring pLP-AmpR was studied parallel with the negative control strain containing pKD46 in the presence and absence of the inducers. In each repetition of the described protocol (a total of eight repetitions), the plasmid pLP-AmpR was eradicated 12 h after induction by anhydrotetracycline and L-rhamnose compared to the control culture; in addition, the number of colonies obtained from the control samples (ranged from 51 to 78 colonies) was not significantly different (Fig. 4). Simultaneously eliminating pLP-AmpR and pScissors-CmR-SCS was unsuccessful, and all samples grew on LB agar containing Amp and Cm (each separately or in combination).

We assessed the function of the landing pad by directly transforming the *tetA* gene flanked by *attB* sites into BL21(DE3) containing pLP-AmpR and plating cells on LB agar supplemented with only tetracycline. Following ten repetitions of the described protocol (cell harvest time: OD600 = 0.5, cell density: 3 × 10^9^ CFU/ml, PCR product concentration: 1 μg), 52 tetracycline-resistant colonies were obtained, all 12 randomly selected colonies were carrying the recombinant plasmid. Results of enzymatic digestion and sequencing from extracted plasmids of multiple tetracycline-resistant colonies confirmed the in vivo gene exchange process. Transformation efficiencies were determined 2×10^8^ to 5.5×10^8^ CFU/μg DNA.

The ICPS method was studied by directly transforming the PCR product into BL21(DE3) containing the mentioned plasmids and plating cells on LB agar supplemented with Amp and Cm. Following ten repetitions of the protocol (cell harvest time: OD600 = 0.5, cell density: 3 × 10^9^ CFU/ml, PCR product concentration: 1 μg), a total of four colonies were obtained, all carrying the recombinant plasmid. To optimize the above protocol, the effect of different parameters, such as cell harvest time, cell density, and PCR product concentration, was investigated during ten repetitions separately for each. According to the number of colonies obtained, the best condition for cell harvest time: OD600 = 0.5 (four colonies), cell density: 2 × 10^9^ CFU/ml (six colonies), and PCR product concentration: 1.5 μg (eight colonies) was determined. Following ten repetitions of the optimized protocol by combining the best condition of each parameter (cell harvest time: OD600 = 0.5, cell density: 2 × 10^9^ CFU/ml, PCR product concentration: 1.5 μg), a total of 12 colonies were obtained, all carrying the recombinant plasmid (Table 3).

**Table 3:** Effect of three evaluated parameters in optimizing the ICPS protocol

Protocol	Cell harvest time (OD600)	Cell density (CFU/ml)	PCR product concentration (μg)	Number of colonies obtained	Average colonies/attempt
ICPS	0.5	3 × 10^9^	1	4	0.4
Optimized ICPS	0.5	2 × 10^9^	1.5	12	1.2

**Fig. 5:**
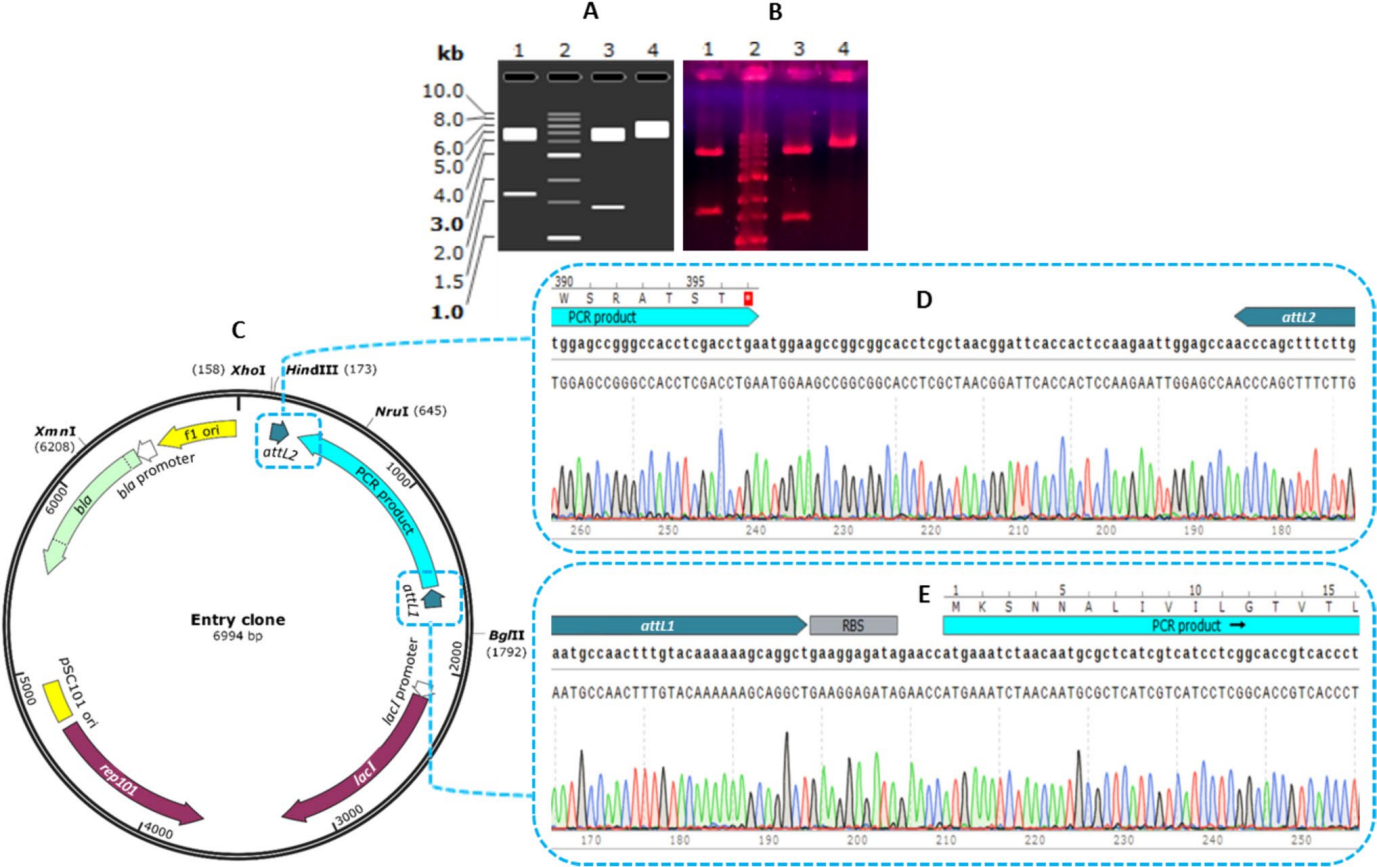
Confirmation of the entry clone by enzymatic digestion and sequencing. **A** In silico agarose gel electrophoresis of the digested recombinant plasmid; 1kb DNA ladder (lane 2). Double digestion by *Xho*I and *Bgl*II yielded two fragments with 5360 and 1634 bp size (lane 1). Double digestion by *Nru*I and *Xmn*I yielded two fragments with 5563 and 1431 bp size (lane 3), and single digestion by *Hin*dIII yielded one fragment with 6994 bp size (lane 4). **B** Obtaining the mentioned results from agarose gel electrophoresis in vitro*.* **C** Schematic map of the recombinant plasmid with recognition sites for *Xho*I, *Bgl*II, *Nru*I, *Xmn*I, and *Hin*dIII. **D** The performed sequencing by universal primers of the T7 terminator and **E** T7 promoter shows the gene was cloned in the right direction

We confirmed the recombinant plasmids by enzymatic digestion and sequencing results (Fig. 5, Supplemental Figs. S1 and S2).

## Discussion

Although many restriction-free cloning techniques are available as alternatives, restriction enzyme–based cloning methods are still widely used. A laboratory habit may be the reason for using these traditional methods. The technical complexity or high cost of alternative methods may be other reasons. Therefore, we aim to introduce a simple and more affordable strategy for routine cloning and expression of a target gene without the expensive reagents and conventional steps of digestion, ligation, and screening.

The ICPS method is based on site-specific recombination, but unlike the Gateway system, bacteria can be directly transformed by PCR products. Therefore, this strategy, on the one hand, simplifies gene cloning by skipping in vitro assembly reactions, and on the other hand, it reduces costs by eliminating in vitro expensive materials such as BP and LR clonase. The Gateway system uses a *CcdB-sensitive strain* to obtain colonies containing recombinant (entry or expression) plasmids and a *CcdB-resistant strain* to propagate non-recombinant (donor or destination) plasmids (Walhout et al. 2000). On the other hand, it involves two steps of bacterial transformation before obtaining the final recombinant vector (Hartley et al. 2000). In the ICPS method, all the requirements to obtain the final recombinant vector are provided in a single strain and require one transformation step. The Gateway technology takes advantage of various destination vectors and several strategies (including *MultiSite* cloning); however, except for the native protein expression plan, in all approaches, additional amino acids are attached to the target protein due to the translation of *attB* (scar) sites (Esposito et al. 2009), which can affect experimental outcomes (Alzari et al. 2006). The ICPS method is designed for native protein expression; *att* sites are placed outside the open reading frame (ORF) using primers that include the Shine-Delgarno element between the *attB1* site and the target gene sequence (Table 4). It should be noted that the Gateway technology and the ICPS have limitations for cloning large genes, as the BP cloning efficiency drops significantly when the targeted DNA length exceeds 2 kb (Marsischky and LaBaer 2004).

**Table 4:** Comparison of Gateway and ICPS methods for routine gene cloning and expression

Cloning method	Gateway	ICPS
Required enzyme (s)	BP and LR clonase (in vitro)	Only BP clonase (in vivo)
Required strain (s)	*CcdB*-*resistant strain for toxic plasmid-propagating and CcdB*-*sensitive strain for final plasmid screening*	Only strain harboring pLP-AmpR and pScissors-CmR for plasmid-propagating, in vivo cloning, and final plasmid screening
Transformation step (s)	Two steps for transforming products of BP and LR reactions	One step for directly transforming PCR products
Cloning plan (s)	Several plans, including with/without scar translation	Only scarless (native protein expression) plan
Required primer (s)	Two primers, including *attB1* primer with/without Shine-Delgarno sequence	Two primers, including *attB1* primer with Shine-Delgarno sequence
Efficiency	High	Low
Cost	Expensive	Cheap

**Fig. 6:**
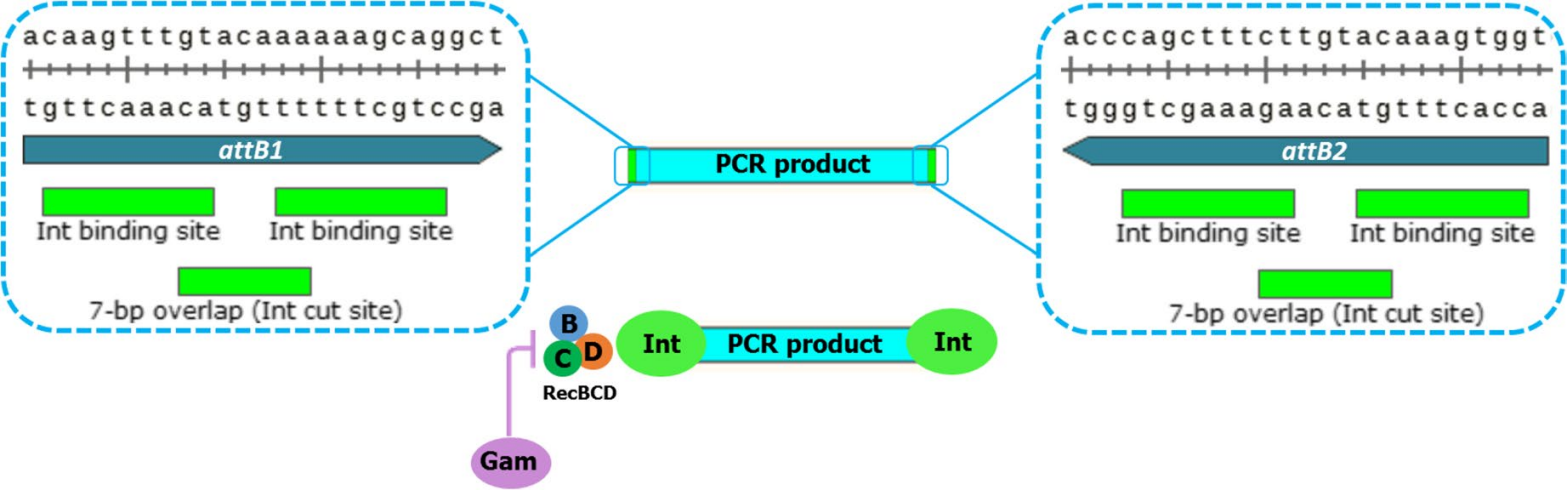
The linear DNA protection from degradation by V (RecBCD) and I (SbcB) host exonucleases. In addition to the role of Gam protein as an exonuclease inhibitor, Int binding sites at both ends of the PCR product may also protect linear DNA

We employed lambda Gam protein as a host-exonuclease inhibitor, allowing direct transformed PCR product to be maintained before *in vivo* recombination (Sharan et al. 2009; Mosberg et al. 2010). Although integrase plays a central role in gene exchange (Katzen 2007), adding Int binding sites at both ends of the PCR product may also protect the linear DNA, similar to the mechanism of gene protein 2 (gp2) from bacteriophage T4 (Petrov et al. 2010) (Fig. 6).

In the past years, plasmid curing from bacteria was difficult, but recent studies have confirmed that CRISPR/Cas9 technology can accelerate this process in many cases. CRISPR-Cas9 can eradicate an antibiotic resistance gene-carrying vector when the plasmid copy number (PCN) is 50–70 per cell (Citorik et al. 2014). In the plasmid curing experiments, we aimed to completely remove the vectors from the cell in a short time (maximum 24 h). Otherwise, this cloning method will be complicated and tedious due to the high number of background colonies**.**

The plasmid pCas9-SSEC could not eradicate pET-21b(+)-LP and pGP1-2-SCS vectors with origins of pBR322 (PCN of 40 ± 0.6) and p15A (PCN of 14-18), respectively (Hochkoeppler 2013). These results may be related to the initial frequency of vectors (Tagliaferri et al. 2020), as pCas9-SSEC can successfully eradicate pKD46-SCS plasmid with the origin of pSC101 (PCN of 5, 6.7 ± 0.2) (Hochkoeppler 2013). Presumably, leaky transcription of the sgRNA from the tightly regulated L-rhamnose promoter is sufficient to eliminate pKD46-SCS since plasmid curing occurred without L-rhamnose induction. In some studies that require an inducible CRISPR/Cas9 system, transcription from both *SpCas9* and sgRNA promoters is under control (Zhao et al. 2016; Lauritsen et al. 2017).

The tetracycline screening resulted in more colonies than the CRISPR and ampicillin screening; therefore, the low efficiency of the ICPS method may be due to the higher metabolic burden or toxicity caused by the pScissors-CmR-based plasmid curing (Cho et al. 2018; Zhang and Voigt 2018; Rouches et al. 2022). However, reducing the cell density to 2 × 10^9^ CFU/ml and increasing the PCR product to 1.5 μg were effective in optimizing the ICPS protocol. On the other hand, simultaneously eliminating pLP-AmpR and pScissors-CmR-SCS was unsuccessful; therefore, after obtaining a recombinant colony by the ICPS method, there is still the additional vector of pScissors-CmR related to the plasmid curing system in the host. In this regard, we are designing a new strategy for the plasmid curing system using I-SceI homing endonuclease. The high efficiency of I-SceI endonuclease in plasmid curing has been confirmed (Volke et al. 2020). Adding the *I-SceI* gene and its cleavage site to the landing pad (LP) instead of SCS can convert the two-plasmid-based ICPS method to a one-plasmid method independent of pScissors-CmR. In the one- plasmid-based ICPS method, Int and IHF proteins replace the PCR product with the LP to form a recombinant (entry) plasmid lacking the I-SceI cleavage site. Subsequently, inducible expression of the I-SceI endonuclease leads to the self-curing of non-recombinant plasmids containing the I-SceI cleavage site.

In conclusion, although the results confirmed the gene replacement and plasmid curing processes, the ICPS method was low efficient. However, we are designing a one-plasmid-based ICPS method by changing other parameters, including using an I-SceI endonuclease instead of an RNA-guided endonuclease for plasmid curing. This initial platform can be the foundation for developing a simple and inexpensive cloning method.

## Supplementary information

ESM 1 (PDF 1292 kb)

## Data Availability

The datasets generated during and/or analyzed during the current study are available from the corresponding author on reasonable request.
