# Supplementary material for: In vivo cloning of PCR product via site-specific recombination in Escherichia coli
Source: Appl Microbiol Biotechnol. 2024 Jun 29;108:400. doi: 10.1007/s00253-024-13239-7 (PMC11217044; doi:10.1007/s00253-024-13239-7)
Supplement: Supplementary file 1 [file 253_2024_13239_MOESM1_ESM.pdf]

Supporting Information

Applied Microbiology and Biotechnology

In vivo Cloning of PCR Product via Site-Specific Recombination in *Escherichia coli*

Moein Aliakbari[1], Ali Asghar Karkhane[1]*

[1] Department of Industrial and Environmental Biotechnology, National Institute of Genetic Engineering and Biotechnology (NIGEB), Tehran, Iran

* Corresponding author

E-mail: karkhane@nigeb.ac.ir

A

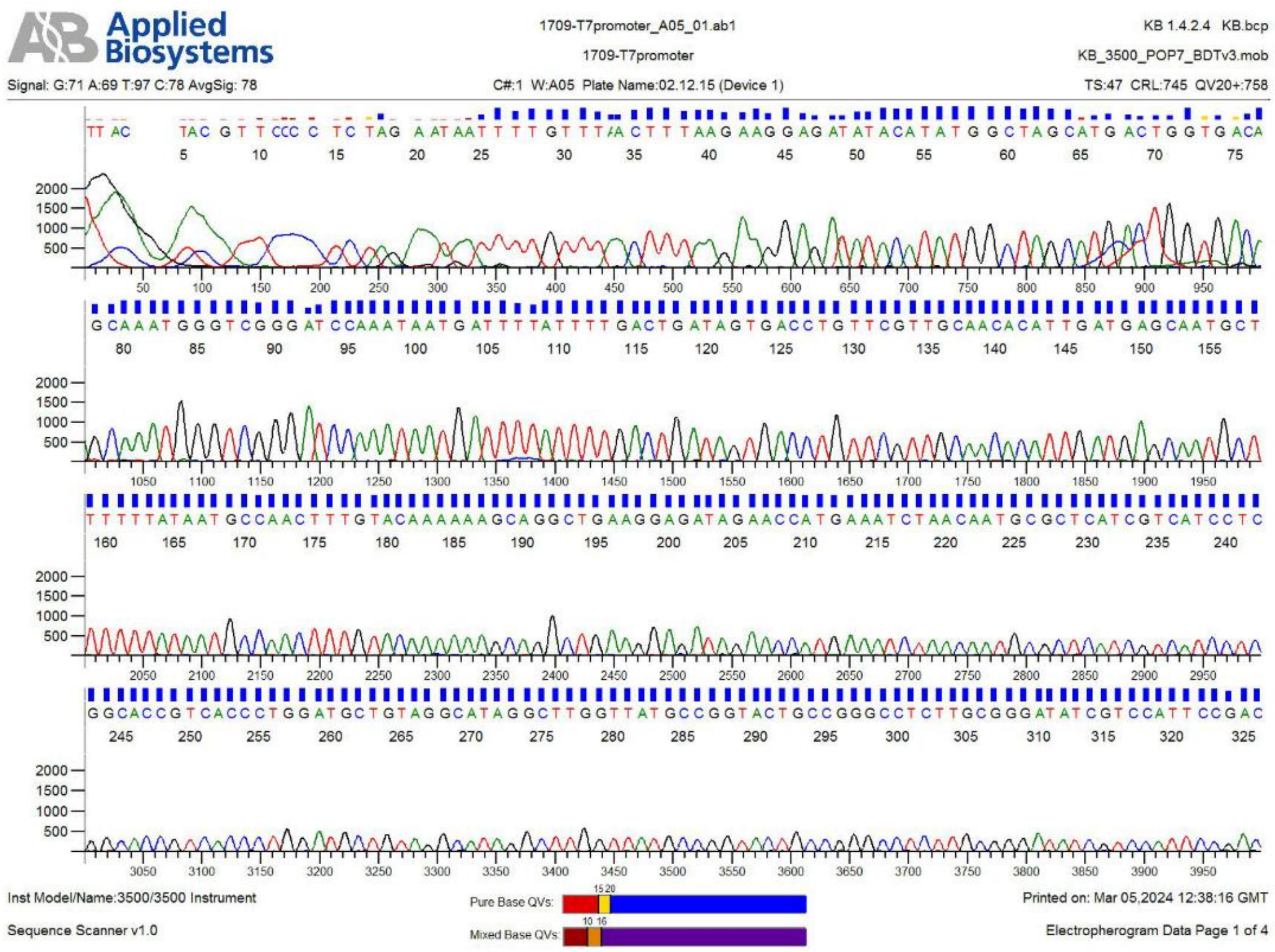

B

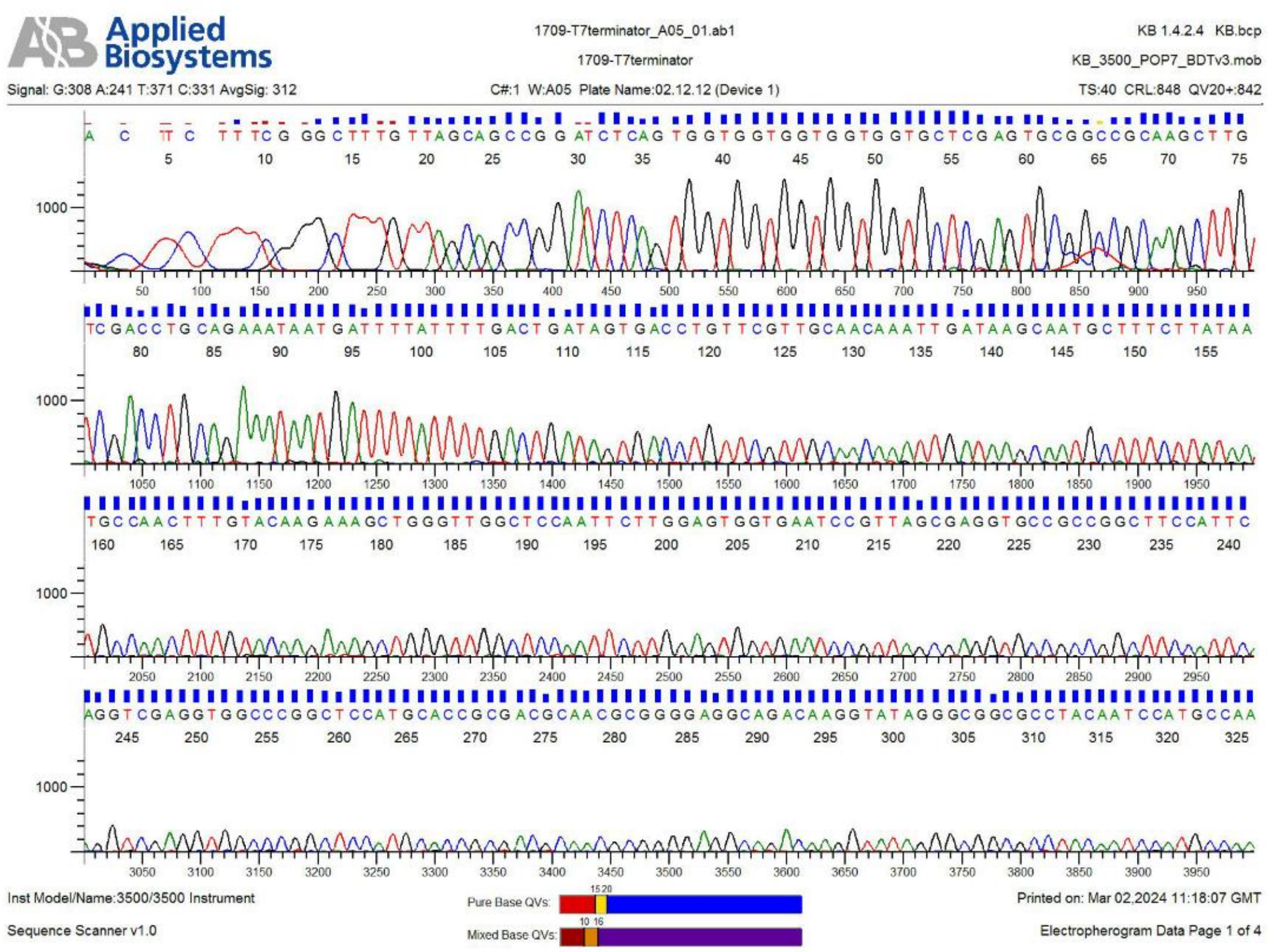

Fig S1. Sequencing chromatogram of the inserted fragment. Related to Fig 5 (A) The performed sequencing with universal primers of the T7 promoter and (B) T7 terminator.

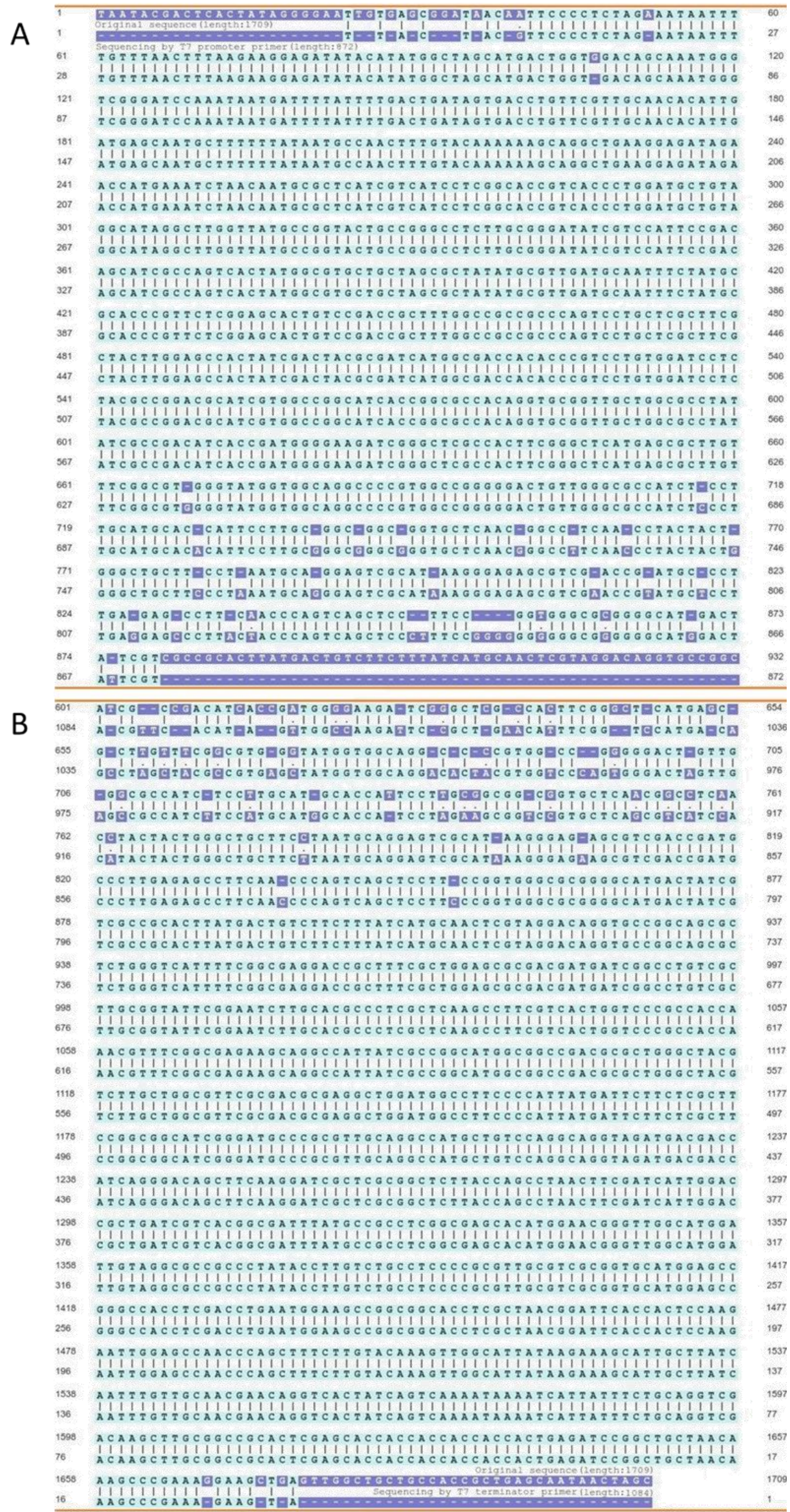

Fig S2. The alignment of sequencing data with the sequence of the *tetA* gene (flanked by *attL* sites). (A) The correct direction of the insert fragment was confirmed by alignment with sequencing data related to the T7 promoter and (B) T7 terminator primers.
